# Perceptions, knowledge, and practices related to oral health in a group of pregnant women: A qualitative study

**DOI:** 10.1002/cre2.823

**Published:** 2023-12-10

**Authors:** Juliana Velosa‐Porras, Nelcy Rodríguez Malagón

**Affiliations:** ^1^ Department of Clinical Epidemiology and Biostatistics Pontificia Universidad Javeriana Bogotá Colombia; ^2^ Centro de Investigaciones Odontológicas CIO, Facultad de Odontología Pontificia Universidad Javeriana Bogotá Colombia

**Keywords:** knowledge, oral health, pregnant women, socioeconomic factors

## Abstract

**Background:**

It is well known that the prevalence of dental caries and periodontal disease is increasing in pregnant women. Dental care is mostly sufficient to prevent oral diseases and perform timely interventions. However, few pregnant women go to the dental office during this period due to a lack of knowledge. The perceptions, knowledge, and oral health practices of pregnant women have been scarcely explored and should be taken into account to propose positive interventions in this population.

**Objectives:**

To identify and explore the perceptions, knowledge, and practices of oral health in a group of pregnant women in Colombia.

**Material and Methods:**

Twenty‐four semistructured interviews were conducted in different areas of Colombia. The interviews were conducted via telephone, and the calls were recorded with the consent of the participants. The recordings were transcribed in Word® and checked for typing and transcription errors. The transcripts were analyzed using a hybrid approach combining inductive and deductive coding. The information was organized and encoded using NVivo12 ®software. We followed the Standards for Reporting Qualitative Research (SRQR) checklist.

**Results:**

Pregnant women reported having good oral health. One barrier mentioned for occupation (Job) was pregnancy. The appearance of the oral cavity has not negatively affected the relationships of pregnant women.

**Conclusions:**

The pregnant women had good oral hygiene habits but did not receive dental check‐ups. Knowledge in this group was limited and diverse. The information given by health professionals should be standardized, and some beliefs should be demystified.

**Relevance to Clinical Practice:**

In general, few pregnant women were aware of bleeding gums during pregnancy. Empower pregnant women to take care of themselves through regular dental check‐ups with the aim of preventing and treating oral diseases. Oral hygiene education and healthy nutritional habits should be intensified during this stage.

**Patient or Public Contribution:**

The participation in the study included a semistructured interview by telephone with the prior consent of the pregnant woman authorizing her involvement and the recording of the interview.

## 1 INTRODUCTION

Oral diseases are generally infectious, and diet and oral care play an important role in the prevention of these diseases. Changes in estrogen and progesterone levels during pregnancy can affect oral health by altering the production, pH and composition of saliva, allowing bacterial colonization. There is also an increase in vascular permeability and a decrease in the response to oral microorganisms, allowing the development of dental caries and periodontal disease, the most common of which is gingivitis (Marrero Fente et al., 2003). Periodontal disease has been reported as a risk factor for preterm labor and low birth weight (Díaz Valdés & Valle Lizama, 2015). To prevent oral disease, pregnancy complications and vertical mother‐to‐child transmission of oral bacteria, WHO has published several guidelines and recommendations on maternal health practices during and after pregnancy (Virtanen et al., 2015). Lack of knowledge about oral diseases among pregnant women has been reported in China and Sudan (El‐Mahdi Ibrahim et al., 2016; Zhong et al., 2015). Education and prevention of oral diseases have been recommended as part of antenatal care programs (El‐Mahdi Ibrahim et al., 2016; Zhong et al., 2015). Similarly, in India (Sajjan et al., 2015), it has been suggested to strengthen oral hygiene practices and develop oral health maintenance strategies for pregnant women (Sajjan et al., 2015).

The Colombian health system (Law 100 of 1993, 1993) operates through mandatory universal insurance with two types of insurance: contributory (financed by payroll contributions) and subsidized for the population without the capacity to pay (financed by tax collection). The Colombian health system (Ley 100 de, 1993) has a primary care plan for pregnant women. This aims to reduce the morbidity and mortality of the mother‐child binomial and oral diseases to improve maternal health (Delgado Gallego, 2007). However, in the IV National Oral Health Study (ENSAB IV), the prevalence of periodontitis in the pregnant group was 37.1% using the CDC‐AAP (American Academy of Periodontology and CDC) classification and 42.1% using the EFP (European Federation of Periodontology) classification. In addition, a prevalence of 59% for dental caries and a prevalence of 77.3% for partial edentulism in the posterior segment were reported (data in press). This suggests that oral diseases are highly prevalent among pregnant women in Colombia, despite the existence of specific programs for this population (Cancino et al., 2018; Ministerio de Salud y Protección Social – Colciencias, 2013).

A Colombian study found that pregnant women living with or under the influence of their partner were more likely to believe that they would lose one tooth per pregnancy (52% and 41.6%, respectively) (Rengifo, 2009). The same study found that the belief that dental care was harmful to the baby was more common among women covered by the National Health Service.

Given the high prevalence of oral diseases and the existence of certain negative beliefs that influence the oral health habits of pregnant women in Colombia, it is important to identify and study the oral health perceptions (how women perceive and understand oral health in relation to pregnancy), knowledge and practices of this group. The results can serve as a basis for the development of proactive interventions to improve the oral health of this population and for future research.

## 2 MATERIALS AND METHODS

### 2.1 Study design

**Figure 1:**
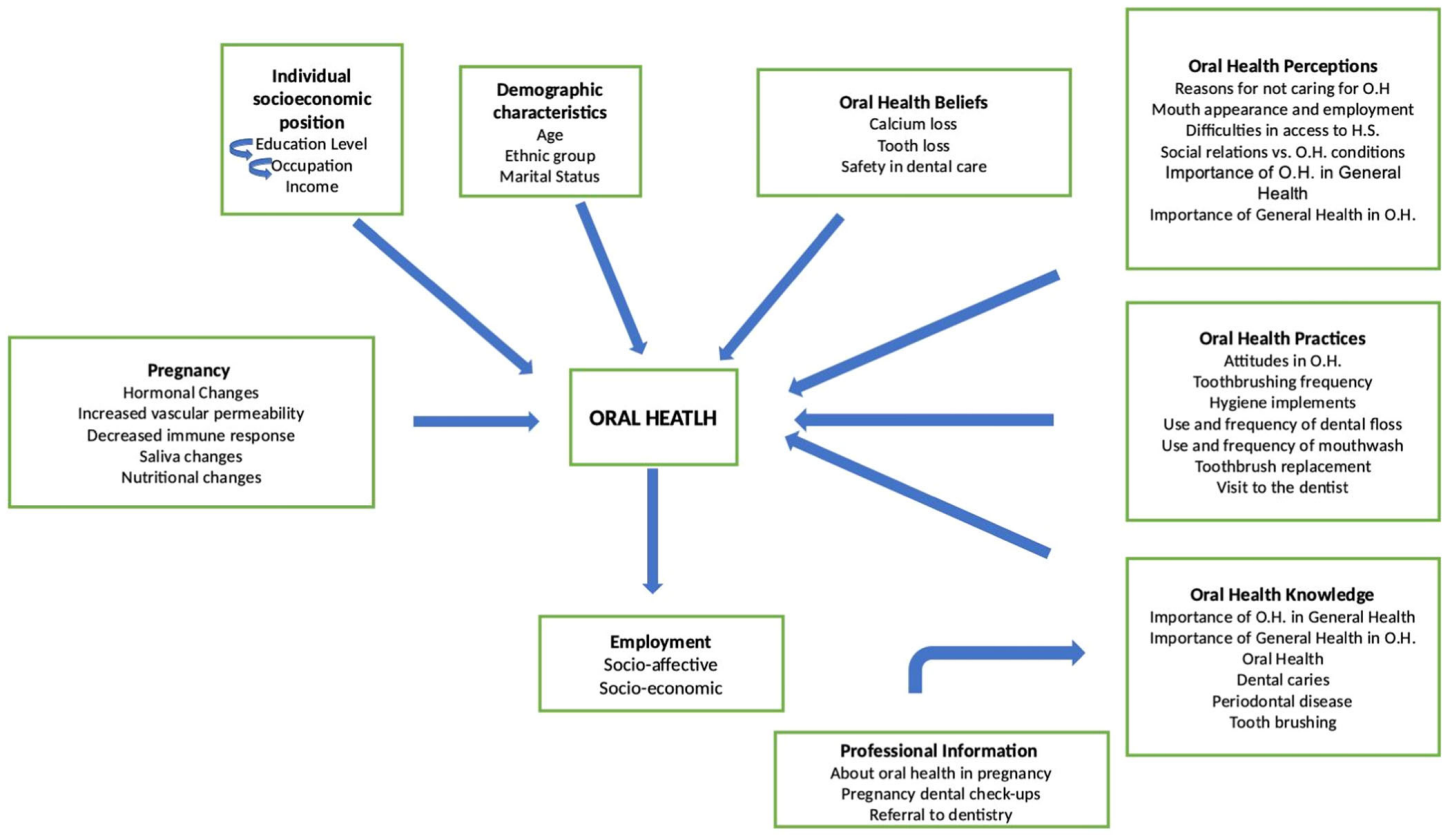
Conceptual model of perceptions, knowledge, and practices in oral health in pregnant women.

This qualitative study had a descriptive phenomenological approach, which allowed the interpretation of the phenomena in depth, explaining the reality of the participants, giving meaning to their experience (De los Reyes Navarro et al., 2020; Leal, 2000). Semistructured interviews were conducted with a group of pregnant women from different areas of Colombia (Figure 1).

### 2.2 Sampling

The study included 24 pregnant women who were selected intentionally by predefined cases, taking into account: age range between 18 and 45 years, between the second and third trimester of gestation, residence in the Departments of Boyacá, Cauca, Antioquia, Cundinamarca, or Bogotá D.C. and attendance at some regional hospitals, departmental health secretariat, or private clinics. The characteristics of the participants are described in Table 1.

**Table 1:** Characteristics of the interviewed pregnant women (*n* = 24).

Participant no.	Age (years)	Education level	Marital status	Regimen	Ocupation	Residence	Monthly household income	Trimester of pregnancy
1	42	University or higher	Married	Contributive	Employee	Bogotá D.C.	1–2 SMLV	Third
2	30	High school completed	Married	Subsidized	Housewife	Combita‐Boyaca	1–2 SMLV	Third
3	20	High school completed	Single	Subsidized	Housewife	Combita‐ Boyaca	<1 SMLV	Second
4	24	Completed elementary school	Single	Subsidized	Housewife	Combita‐ Boyaca	<1 SMLV	Second
5	22	High school completed	Free Union	Subsidized	Student	Combita‐ Boyaca	<1 SMLV	Second
6	29	University or higher	Married	Contributive	Employee	Zipaquirá‐ Cundinamarca	>2 SMLV	Second
7	19	High school completed	Free Union	Subsidized	Housewife	Totoró‐ Cauca	<1 SMLV	Third
8	18	High school completed	Single	Subsidized	Housewife	Combita‐ Boyaca	<1 SMLV	Second
9	30	Completed elementary school	Single	Subsidized	Housewife	Chiquinquirá‐ Boyaca	<1 SMLV	Third
10	41	Completed elementary school	Single	Subsidized	Unemployed	Combita‐Boyaca	<1 SMLV	Second
11	39	University or higher	Single	Contributive	Employee	La Sierra ‐ Cauca	1–2 SMLV	Second
12	22	Technician or technologist	Free Union	Contributive	Unemployed	Bogotá D.C.	1–2 SMLV	Third
13	36	University or higher	Casada	Contributive	Employee	Medellín ‐ Antioquia	>2 SMLV	Second
14	28	University or higher	Free Union	Contributive	Employee	Bogotá D.C.	>2 SMLV	Second
15	44	Completed elementary school	Free Union	Subsidized	Housewife	Combita‐ Boyaca	<1 SMLV	Third
16	20	High school completed	Free Union	Subsidized	Housewife	Combita‐ Boyaca	<1 SMLV	Second
17	36	University or higher	Free Union	Contributive	Employee	Bogotá D.C.	>2 SMLV	Second
18	40	University or higher	Free Union	Contributive	Employee	Bogotá D.C.	>2 SMLV	Second
19	38	High school completed	Free Union	Contributive	Housewife	Bolivar ‐ Cauca	1–2 SMLV	Third
20	39	High school completed	Single	Subsidized	Unemployed	Arcabuco‐ Boyaca	<1 SMLV	Third
21	33	Completed elementary school	Married	Subsidized	Housewife	Arcabuco‐ Boyaca	<1 SMLV	Third
22	33	University or higher	Married	Contributive	Employee	Madrid‐ Cundinamarca	>2 SMLV	Third
23	31	University or higher	Married	Contributive	Employee	Bogotá D.C.	>2 SMLV	Second
24	30	High school completed	Single	Contributive	Employee	Bogotá D.C.	1–2 SMLV	Third

### 2.3 Information collection

The interviews were conducted between May and November 2020, and all were conducted by telephone. The calls were recorded with the consent of each pregnant woman. The questions of the semistructured interviews were open. Some were formulated by the main researcher, and others were adapted from the questionnaire of the IV National Oral Health Study (ENSAB IV). The questions focused on addressing personal experiences and meanings regarding their state (pregnancy), perceptions, knowledge and aspects related to oral health. Some of the questions were as follows: During your daily work, do you spend time caring for your mouth? What are the reasons why you do not spend time taking care of your mouth at the end of the day? Have you ever lost the opportunity to get a job because of the look of your mouth? Have you sought dental treatment during your pregnancy? Do you think that you currently need dental treatment? Have you been educated about how to avoid dental problems during pregnancy? Do you think that a tooth is lost for every pregnancy? Do you think the baby steals your calcium? What are the reasons why we should brush our teeth?

The recordings were transcribed in Word and checked for typing and transcription errors. Each interview lasted approximately 50 min.

### 2.4 Analysis of information

The demographic characteristics of the pregnant women were summarized using descriptive statistics. The transcripts were analyzed using a hybrid approach combining inductive and deductive coding (Martínez Pérez, 2009; Fereday & Muir‐Cochrane, 2006). In this process, the first step was the deductive coding created from the scheme of semistructured interviews. The codebook was reviewed by the researcher using inductive coding. The list of codes was reduced to categories to create the final codebook with which the transcripts were coded.

The information was organized and encoded using NVivo12 software. Topics related to oral health perceptions, knowledge and practices among pregnant women were analyzed. The study found saturation of the information when thematic categories were repeated and there was no need to add new codes during the analysis (Burnard et al., 2008). The reporting method used was The Standards for Reporting Qualitative Research (SRQR; Supporting Information: File 1).

## 3 RESULTS

The 24 pregnant women included in the study had a median age of 30.5 years, with little variability between trimesters of pregnancy. The most common levels of education were university and high school. In terms of place of residence, 11 lived in the Department of Boyacá, 7 in Bogotá, and 3 in Cauca (Table 2).

**Table 2:** Sociodemographic characteristics of the interviewed pregnant women.

Sociodemographic characteristics	
Age (years)	
Mean	31 (SD: 8.04)
Median	30.5

	*n* (%)
Age group	
15–24	7 (29.1)
25–34	8 (33.3)
35–45	9 (37.5)
Marital status	
Single	8 (33.3)
Married	7 (29.1)
Free union	9 (37.5)
Monthly household income	
Less than 1 SMLV (COP$877,803)	11 (45.8)
Between 1 and 2 SMLV	6 (25)
More than 2 SLMV	7 (29.1)
Education level	
Completed elementary school	5 (20.8)
High school completed	9 (37.5)
Technician or technologist	1 (4.1)
University or higher	9 (37.5)
Ocupation	
Housewife	8 (33.3)
Employee	9 (37.5)
Unemployed	5 (20.8)
Student	1 (4.1)
Independent	1 (4.1)
Residence	
Bogotá D.C.	7 (29.1)
Boyacá	11 (45.8)
Cauca	3 (12.5)
Cundinamarca	2 (8.3)
Antioquia	1 (4.1)
Regimen	
Subsidized	12 (50)
Contributive	12 (50)
Trimester of pregnancy	
Second trimester	13 (54.1)
Third trimester	11 (45.8)

The educational level of the pregnant housewives or unemployed was completed primary or high school, and the monthly household income was <1 SMLV. Among the participants who were employed and did not have favorable working conditions, the household income was between 1 and 2 SMLV.

### 3.1 Perception of oral health

The unemployed women and housewives were in a vulnerable position due to the lack of paid employment. Housewives mentioned that pregnancy was an obstacle to work. Some of the employed women had long working hours. Some of the expressions used by the pregnant women in relation to the issue were as follows:

…what comes out of me, whatever has to be done, I do to find sustenance… (Pregnant woman 10)

…When I have to close up, I am returning home at almost 9:30–10:00 p.m. … I am very tired… (Pregnant woman 24)

Among the participants who were employed and did not have favorable working conditions, the household income was between 1 and 2 SMLV. Seven people reported poor or fair oral health.

### 3.2 Knowledge of good oral health

As far as knowledge about good oral health is concerned, the results are mixed. In most cases, the absence of tooth decay seems to be an aspect of good oral health. It was found that at lower levels of education, the terminology used to define oral health was more colloquial. Expressions such as the following illustrate this situation:

…not having black teeth, suddenly pitted, pain in the gums, inflammation, bad smell… (Pregnant woman 12)

…the teeth do not have any caries theme … the bite … that the gums are healthy … not having bad breath, some different coloration in your mouth… (Pregnant woman 23)

### 3.3 Knowledge about the causes of oral disease

All participants mentioned a sweet tooth and/or poor oral hygiene as a cause of tooth decay; one also highlighted the importance of childhood education, and another also mentioned genetic predisposition. Some stated:

…it is the neglect of one … it is a matter of education. They do not instill in one in childhood that care… (Pregnant woman 20)

…we have a certain genetic prevalence that the bone of the tooth is less strong and protected… (Pregnant woman 23)

Only three participants with a university education knew about periodontal disease; they defined it as an inflammatory process caused by hormonal changes during pregnancy or by an infectious process. In general, few subjects knew about gum bleeding during pregnancy, although several participants stated that they had experienced this change.

### 3.4 Consequences of suboptimal oral health

The appearance of the oral cavity has not negatively affected relationships with other people for the majority of pregnant women, except for two who expressed that the lack of teeth has led to bullying problems, including nicknames and discrimination, as expressed in the following:

…as I have prostheses, this is something that helps me, but if I did not have prostheses then yes, it would be terrible because already, they discriminate against you… (Pregnant woman 10)

One participant explained that she had lost a job because of the appearance of her teeth and that her poor oral health was a barrier to employment opportunities.

…I think so, that is very important when applying for certain types of employment, where personal presentation influences a lot … Well, they don't tell you directly, but you know that this is so… (Pregnant woman 20)

The two pregnant women mentioned above had these circumstances in common: they were unemployed, had a family income of less than 1 SMLV, belonged to the subsidized regime and were single. In terms of education, one had completed high school and the other had only completed primary school. Given these circumstances, it is possible to observe the extent of oral health in psychosocial circumstances and in employment, and this in socioeconomic position.

### 3.5 Oral health in pregnancy

Pregnant women reported that pregnancy had an effect on oral health, such as increased tooth sensitivity, loss of calcium, increased bleeding in the gums, and decalcification of the teeth. Two participants mentioned that this information had been provided by the gynecologist and a periodontist. When asked what type of disease could affect oral health, they mentioned reflux, diabetes, gastrointestinal disease, or liver problems, but they could not identify what oral manifestations could occur.

Two common beliefs about pregnancy and oral health are tooth loss and calcium loss. All pregnant women, whether they agreed or disagreed with the statement “the baby is stealing my calcium,” reported supplementing their diet with calcium (Pregnant women 15, 12, 9, 24, 3, 11, 6, 1, 22, 21, 18, 17, 16, 14, 10, 20, 13). When asked about the belief that teeth are lost with every pregnancy, all participants said that this was not true (“it's a myth” or “I don't think so”). One pregnant woman said that the nurse at one of the antenatal clinics told her that she could lose her teeth because of pregnancy (Pregnant woman 18).

All participants stated that it was everyone's responsibility to maintain good oral health, which included brushing teeth three times a day and having regular check‐ups. One participant stated that it was the responsibility of the state to provide good and safe dental services in health centers and in the private sector: biosafety and quality of treatment was a barrier to regular dental check‐ups and therefore inadequate oral health.

### 3.6 Oral health practices

#### 3.6.1 Brush your teeth

The entire study group reported brushing their teeth between one and three times a day. They used toothbrushes and toothpaste. The amount used varied from the size of a bean to covering all the bristles of the brush and repeating the application. The use of dental floss was reported less frequently, with the majority using it only once a day, preferably at night after brushing. Two participants reported that they did not floss because of bleeding and pain. Rinsing with mouthwash was only done occasionally because of the cost, because it affects the baby, or because of the sensation it leaves in the mouth. This is how they expressed it:

…the economic condition depends, when there are possibilities of buying it and getting it in the market, one uses it, but when money is scarce, one cannot get it… (Pregnant woman 20)

All participants said that they had the time and flexibility in their daily work to take care of their mouths. One mentioned that she sometimes felt reluctant because of the nausea caused by pregnancy.

…of course, but no, I can't do that right now. I can't brush because it makes me nauseous. (Pregnant woman 20)

Regarding the time of day that they dedicated to brushing, all participants said that they did it in the morning and in the evening before going to bed, except for one woman who said that she did not do it in the morning because of the nausea caused by brushing. Two participants also stated that they were aware that brushing was superficial care because they did not use floss or mouthwash.

…at night I only bathe my mouth no more. (Pregnant woman 20)

Regarding tooth brushing, all participants said that it allowed them to have and maintain adequate oral health, including no cavities, bleeding and bad breath. They also said that taking care of their teeth was a matter of personal image as well as hygiene.

Participants said they replaced their toothbrushes when the bristles opened or fell out, which could take 1–6 months. One participant said that toothbrushes should be replaced at least every 3 months because the brush loses its effectiveness; another participant said that a dentist recommended replacement due to contamination.

### 3.7 Nutritious habits

All the participants reported an increase in the frequency of eating and in the consumption of fruit and water during their pregnancy; six also reported an increase in the consumption of sweets and carbohydrates such as potatoes and cassava, others in flour such as bread or biscuits. This increased consumption of carbohydrates increases the risk of tooth decay in this population. An example of what was expressed was as follows:

…I have to eat for 2 … Let's say this, bread and cookies, I am very fascinated… (Pregnant woman 9)

### 3.8 Assistance and experience in dental care

All pregnant women were linked to the antenatal care program and reported having regular antenatal check‐ups, either in person or online, from around the sixth week of pregnancy. Behavior varied with regard to the three dental check‐ups. Less than half of the women had a single check‐up.

The time since the last dental check varied from 1 week to 2 years. For 14 participants, this last check‐up was part of the basic care plan for pregnant women. Five participants had not had a dental check‐up during pregnancy, for reasons such as not needing one, COVID‐19, fear of infection, or because the clinics were closed. None of them reported any problems getting an appointment. Here is how they expressed it:

…No, the truth is that I have not gone because of COVID‐19. (Pregnant woman 11)

The most common places for dental care were the health center and the health promotion unit (EPS). The reasons for attending included referral as part of the primary care plan, toothache and oral hygiene, and already being treated.

Participants reported that the information they received included attending three dental check‐ups. However, they did not receive a referral for a dental consultation at the various antenatal medical check‐ups.

In terms of satisfaction with the dental care they received, most participants said they were satisfied for various reasons, such as the care itself, the treatment, the friendliness, the process, not having to go back for the same reason, the ease of getting an appointment, the follow‐up, and because they had been told about the risks of poor oral health during pregnancy. Some examples:

…It was not abrupt or anything delicate, it covered the hole… (Pregnant woman 24)

…because they explained to me that the risks during pregnancy… (Pregnant woman 8)

## 4 DISCUSSION

The results of this qualitative study show the perceptions, knowledge, and practices related to oral health among a group of pregnant women in Colombia living in the Departments of Boyacá, Cauca, and Antioquia and the city of Bogotá.

The majority of pregnant women perceived themselves to have good oral health and recognized the importance of daily brushing and regular dental visits. They also mentioned the importance of education to develop good oral habits.

When knowledge of different aspects of oral health was assessed, it was found to be limited and varied among the study participants. An example of this is the lack of information about periodontal disease; only three pregnant women with a university degree or higher demonstrated this knowledge. This suggests that knowledge of oral health is partly dependent on schooling, in addition to education provided by dentists and doctors. The results highlight the need for standardized training for professionals to provide information to all pregnant women. These results are consistent with those found in other studies. In China (Wu et al., 2014), 73.9% of women had little knowledge of the possible relationship between pregnancy and oral health. Similar results were found in Saudi Arabia in 2015 (Al‐Swuailem, 2015), where 91.1% were unaware of the link between periodontal disease and preterm birth. In India in 2015 (Sajjan et al., 2015) and 2013 (Avula et al., 2013), 78% did not know that periodontal disease is common during pregnancy, 66% had no knowledge about periodontal disease, and 87.2% had never heard of the link between oral health and adverse pregnancy outcomes. In China in 2015 (Zhong et al., 2015), pregnant women were found to be unaware that dental check‐ups (37%) during the second trimester (18%) were preventive measures. None of the women were aware of the potential consequences of not treating periodontal disease. In India (Gambhir et al., 2015), a systematic review of the literature found that health knowledge and awareness among pregnant women was low. Most women were unaware of the consequences of neglecting oral hygiene during pregnancy.

In general, when the knowledge of this group of pregnant women was assessed, it was found to be based on experience and that none had received adequate and complete information on the topics studied. Therefore, it is imperative to increase, reinforce and empower pregnant women through oral health education.

In terms of beliefs about pregnancy and oral health, pregnant women reported that it was not true that one tooth is lost with each pregnancy, but half of the women believed that their babies would steal their calcium. These findings support those of other authors. In Colombia in 2009, Rengifo (Rengifo, 2009), found that 70% of women said that the baby steals calcium from the mother's teeth. In the United Arab Emirates (Hashim, 2012) in 2012 and Saudi Arabia in 2015 (Al‐Swuailem, 2015), 44.4% and 53.7% of participants, respectively, stated that a woman loses one tooth for each baby. In Saudi Arabia, only 7% of women knew that the fetus does not receive calcium from the mother's teeth.

Satisfaction with dental care plays an important role in this psychosocial context, where professionalism, quality of care, treatment success, and absence of symptoms influence women's decisions to seek dental care. This contributes to tooth loss, which leads to discrimination in their daily lives, including in the workplace. Studies in Greece (Dinas et al., 2007), the United States (Al Habashneh et al., 2005), the United Arab Emirates (Hashim, 2012), Saudi Arabia (Al‐Swuailem, 2015), Australia (George et al., 2013a), and Brazil (Krüger et al., 2015), found that between 20.9% and 58.3% of the women surveyed attended a dental check‐up during pregnancy. Regarding the reasons for consultation, this study found that toothache and already being treated were the main reasons. These results are consistent with those reported in other studies: the main reason for consultation was dental pain in 53.4% (Dinas et al., 2007), 32.8% (Hashim, 2012), and 47.6% (Krüger et al., 2015) of patients. In Saudi Arabia (Al‐Swuailem, 2015), 52% periodontal disease accounted for 52% of the dental needs of pregnant women. In Australia (George et al., 2013a), 29.2% cited reassurance of care during consultation, cost and lack of time as the main barriers to dental care. In Brazil (Krüger et al., 2015), 28% of pregnant women reported difficulties in obtaining dental care, 51.5% reported that dentists refused to see them, and 30% received incomplete treatment, leaving dental problems unresolved. This confirms that more than half of women, regardless of their geographical location, did not visit a dentist during pregnancy, and most of them used health services when they had dental pain. Efforts should be made to educate pregnant women about oral health, especially self‐care, and the importance of attending dental check‐ups during pregnancy to prevent problems.

According to the structure of the health system, which affects access to health services and programs, all pregnant women were informed that they had to attend three dental check‐ups during pregnancy. Unfortunately, these referrals were not given, they were not clear, and the importance of dental care was not explained. As a result, women generally did not follow these guidelines due to ignorance. The pregnant women who came for prepregnancy treatment for pain or bleeding were aware of the need to attend a dental check‐up. Providing clear information will empower women about their oral health, so the majority would follow the guidelines. In Australia in 2013 (George et al., 2013b), only 10% of women surveyed received information about oral health care during pregnancy through pamphlets, antenatal care and dentists. In contrast, India in 2013 (Avula et al., 2013) and Kuwait in 2005 (Honkala & Al‐Ansari, 2005) reported that no pregnant women were referred to a dentist or received information about oral health care during pregnancy.

Regarding oral hygiene habits, all participants reported daily tooth brushing, with most brushing three times a day, although the duration and frequency of brushing is influenced by nausea and vomiting. In this practice, the elements that are not lacking are the toothbrush and the toothpaste. However, the use of dental floss is influenced by bleeding from the gums and is a habit that should be maintained regardless of the consequences. The use of mouthwash is intermittent and determined by economic resources, which shows that the context in which the pregnant woman is immersed can limit access to this basic element of adequate oral hygiene. In Kuwait in 2005 (Honkala & Al‐Ansari, 2005), 64% of women reported brushing their teeth more than once a day and 94% at least once a day. In Nepal in 2018 (Lubon et al., 2018), pregnant women reported using a toothbrush and toothpaste once a day before breakfast as their usual practice, contrary to what was found in focus groups where women had stated that the norm in their community was to brush their teeth twice a day or more. The reasons they gave for oral hygiene were to reduce the risk of tooth decay, general hygiene (disease prevention) and to prevent infection. In terms of barriers to oral hygiene, the main barrier was access to toothbrushes and toothpaste.

The behaviors found in this study reflect the attitudes of the participants. Oral health education should be promoted to improve these unhealthy behaviors, including nonattendance at dental check‐ups, frequency of brushing, use of floss, rinsing and toothbrush replacement.

## 5 CONCLUSION

This study was conducted on a group of pregnant women from different regions of Colombia. The sample was selected on the basis of convenience, due to the design of the study and the circumstances of the COVID‐19 pandemic at the time the information was collected and taking into account the availability of facilities for participation. Therefore, there was a group of women representing each of the sites contacted. Another limitation encountered was the way in which the semistructured interviews were carried out, which were conducted by telephone, which did not allow us to observe the nonverbal language of the pregnant women. This study found that oral health care is a daily activity among the participants, unlike attending dental check‐ups during pregnancy, which was influenced by confusing information given during antenatal check‐ups, income, satisfaction with dental care and fear of COVID‐19. Knowledge about oral health and oral diseases is limited and variable. Based on the results of this study, it is recommended that all pregnant women with characteristics similar to those included in this study be educated about oral health, and that standards be developed for professionals to provide information in a clear, simple and effective manner. It is advisable to empower pregnant women to take care of themselves through regular dental check‐ups to prevent and treat oral diseases, and to increase education on oral hygiene and healthy eating habits.

A preprint has previously been published (Velosa‐Porras & Malagón, 2023).

## AUTHOR CONTRIBUTIONS

Juliana Velosa‐Porras participated in the design of the study, data collection, data analysis, and writing of the article. Nelcy Rodríguez Malagón participated in the design of the study and writing of the article.

## CONFLICT OF INTEREST STATEMENT

The authors declare no conflict of interest.

## ETHICAL APPROVAL

The project was approved by the ethics committee of the San Ignacio University Hospital (FM‐CIE‐0500‐20; FM‐CIE‐0005‐19). All procedures were performed in accordance with the guidelines of the Declaration of Helsinki. Written Informed consent was obtained from all participants.

## Supporting information

Supporting information.

## Data Availability

The data sets generated and/or analyzed during this study are not publicly available because they are interviews with sensitive information but can be obtained from the corresponding author upon reasonable request.
