# Supplementary material for: Perceptions, knowledge, and practices related to oral health in a group of pregnant women: A qualitative study
Source: Clin Exp Dent Res. 2023 Dec 10;10(1):e823. doi: 10.1002/cre2.823 (PMC10860550; doi:10.1002/cre2.823)
Supplement: Supplementary file 1 — Supporting information. [file CRE2-10-e823-s001.docx]

Supplementary File 1.

**[Standards for Reporting Qualitative Research: A Synthesis of Recommendations](https://journals.lww.com/academicmedicine/Fulltext/2014/09000/Standards_for_Reporting_Qualitative_Research__A.21.aspx)**

O’Brien, Bridget C.; Harris, Ilene B.; Beckman, Thomas J.; Reed, Darcy A.; Cook, David A.

Academic Medicine89(9):1245-1251, September 2014.

doi: 10.1097/ACM.0000000000000388

| **No.** | **Topic** | **Item** |
| --- | --- | --- |
| S1 | Title | OK. Page 1 |
| S2 | Abstract | OK. Page 1 |
| S3 | Problem Formultaion | OK. Page 2 |
| S4 | Purpose or reserach question | OK. Page 4 |
| S5 | Qualitative approach and research paradigm | OK. Page 4 |
| S6 | Researcher characteristics and reflexivity | OK. Page 5 |
| S7 | Context | Ok. Page 4 |
| S8 | Sampling strategy | OK. Page 4 |
| S9 | Ethical issues pertaining to human subjetcs | OK. Page 4 |
| S10 | Data collection methods | OK. Page 5 |
| S11 | Data collection instruments and technologies | OK. Page 5 |
| S12 | Units of study | OK. Page 5 |
| S13 | Data processing | OK. Page 5 |
| S14 | Data analysis | OK. Page 6 |
| S15 | Techniques to enhance trustworthiness | OK. Page 6 |
| S16 | Synthesis and interpretation | OK. Page 6 |
| S17 | Links to empirical data | OK. Page 6 |
| S18 | Integration with prior work, implications, transferability and contributions to field | OK. Page 14 |
| S19 | Limitations | OK. Page 19 |
| S20 | Conflicts of interest | OK. Page 19 |
| S21 | Funding | OK. Page 19 |

Standards for Reporting Qualitative Research (SRQR)a

Copyright © 2023 by the Association of American Medical Colleges

2
